# Anoikis-related genes predicts prognosis and therapeutic response in renal cell carcinoma

**DOI:** 10.1080/07853890.2025.2548042

**Published:** 2025-08-19

**Authors:** Lizhi Zhou, Hengcheng Lu, Bin Fu, Jianhan Fu

**Affiliations:** a Jiangxi Provincial Key Laboratory of Urinary System Diseases, Department of Urology, The First Affiliated Hospital, Jiangxi Medical College, Nanchang University, Nanchang, Jiangxi, P.R. China; b Postdoctoral Innovation Practice Base, The First Affiliated Hospital, Jiangxi Medical College, Nanchang University, Nanchang, P.R. China; c Cardiovascular Research Institute of Jiangxi Province, Jiangxi Provincial People’s Hospital, The First Affiliated Hospital of Nanchang Medical College, Nanchang, Jiangxi Provincial, P.R. China; d Department of Urology, Sun Yat-sen Memorial Hospital, Sun Yat-sen University, Guangzhou, P.R. China

**Keywords:** Anoikis, renal cell carcinoma, metastasis, MMP9

## Abstract

**Background:**

Metastasis represents the primary cause of cancer-related mortality, with a high incidence observed in renal cell carcinoma (RCC). Anoikis, a specialized form of apoptosis, plays a crucial role in preventing displaced cells from adhering to new extracellular matrices (ECM), thus inhibiting their aberrant growth. Notably, cancer cells, especially metastatic ones, exhibit resistance to anoikis. However, the exact mechanisms of anoikis resistance in RCC are not well understood.

**Methods:**

This study integrates bioinformatics, single-cell RNA sequencing and experimental validation to investigate the role of anoikis-related genes (ARG) in RCC, with a focus on MMP9. RNA-seq data from 518 RCC patients and 71 healthy controls (TCGA-KIRC) and external validation cohorts (E-MTAB-1980, GSE22541) were analyzed to construct an ARG-based prognostic model. Single-cell RNA sequencing (scRNA-seq, GSE159115) was used to assess tumour heterogeneity, while *in vitro* experiments in RCC cell lines validated MMP9’s impact on anoikis resistance, migration and invasion.

**Results:**

We collected all RNA-seq and single-cell RNA-seq (scRNA-seq) data from multiple online databases and utilized these datasets to develop a novel ARG-based prognostic model called ARGs. Using Cox regression and machine learning, our model achieved a 5-year area under curve (AUC) of 0.79, surpassing existing models in predictive performance. Enrichment analysis revealed distinct immune and metabolic landscapes between ARGs high- and low-risk groups. At the single-cell level, tumour cells were categorized based on ARG expression, revealing heterogeneous anoikis resistance mechanisms. MMP9 was identified as a key prognostic gene (HR = 1.5, *p* = 0.016) associated with anoikis resistance and RCC metastasis. Functional assays confirmed that MMP9 knockdown increased anoikis by 59% and significantly reduced wound-healing migration by about 30% and transwell invasion by 50%, reinforcing its role in RCC progression.

**Conclusions:**

Targeting anoikis-related genes, particularly MMP9, enhances anoikis sensitivity and reduces RCC invasiveness, offering a potential therapeutic strategy to mitigate metastasis and improve clinical outcomes.

## 1. Backgrounds

Renal cell carcinoma represents one of the most common malignant tumours in the urinary system and leading to around 180,000 deaths per year globally [1]. Among its various subtypes, clear cell renal cell carcinoma (ccRCC) is the most aggressive, accounting for 70%-80% of all RCC cases [2]. While early-stage RCC can be effectively treated through surgical resection or ablation techniques, metastatic RCC remains largely incurable, with a 5-year survival rate of only 12%, compared to 75% for RCC overall [3–5]. These statistics underscore the critical need to explore the mechanisms underlying RCC invasion and metastasis, which are crucial for improving patient outcomes and guiding treatment strategies. For localized RCC, treatment options include partial or radical nephrectomy, as well as minimally invasive techniques such as cryoablation and radiofrequency ablation [6]. However, once RCC becomes metastatic, treatment options become limited, primarily relying on systemic therapies such as immune checkpoint inhibitors and tyrosine kinase inhibitors (TKIs) [7]. Although these treatments have improved patient survival, many patients ultimately develop resistance, and the prognosis remains poor, which highlights the necessity for novel therapeutic strategies.

One critical feature of cancer metastasis is the ability of tumour cells to survive and proliferate outside their native microenvironment. Normally, cellular adhesion and interactions with the extracellular matrix (ECM) are essential for cell survival [8]. Anoikis, a specialized form of programmed cell death, occurs when cells lose their connection to the ECM [8,9]. Typically, anoikis efficiently eliminates ectopic cells, preventing their growth on inappropriate tissues and organs [10]. During the process of anoikis, multiple signalling pathways are triggered, such as those mediated by integrins, growth factor receptors and apoptosis-related mechanisms. These pathways collectively drive apoptosis, which is characterized by DNA fragmentation, mitochondrial dysfunction, cytoskeletal reorganization and the activation of caspases [11,12]. Nevertheless, cancer cells can evade anoikis, allowing them to survive, disseminate from the primary site to distant organs and establish metastatic foci [9,13]. Metastasis is the primary cause of death in RCC patients, with anoikis resistance being a crucial characteristic of metastatic cancer [14,15]. Several key pathways might contribute to anoikis resistance, including the PI3K/Akt pathway, which suppresses pro-apoptotic proteins such as BIM and BAX to prevent caspase activation [16]. Similarly, the MAPK/ERK pathway promotes cytoskeletal reorganization and enhances integrin-mediated survival signalling, allowing detached cells to evade apoptosis [17]. Additionally, focal adhesion kinase (FAK) signalling plays a central role by maintaining integrin engagement and activating downstream pro-survival pathways [18]. However, the mechanisms underlying anoikis resistance in RCC remain poorly understood. With the rapid advancement of bioinformatics technologies, it is now possible to systematically analyze sequencing data to identify prognostic anoikis-related genes, evaluate their potential to predict immunotherapy and TKI responses, and explore their role in RCC heterogeneity at the single-cell level.

Given the significance of anoikis resistance in metastatic RCC progression, targeting and modulating this process may offer a novel therapeutic strategy. Previous studies have indicated that the matrix metalloproteinase (MMP) family, which is overexpressed in nearly all cancer types [19,20], including RCC, represents a promising therapeutic target [21]. MMP9, ranking among the top five anoikis-related genes in differentially expressed genes in metastatic ccRCC has been implicated in cancer invasion, metastasis and upregulation across various tumour types [22–24]. Several cancers, such as hepatocellular carcinoma, prostate cancer, and head and neck squamous cell carcinomas, have exhibited altered apoptosis resistance and invasion through the regulation of MMP9-related signalling pathways [25–27].

While MMP9 contributes to maintaining the malignant phenotype of RCC progression, its role in anoikis resistance remains largely unexplored. By degrading ECM components, releasing pro-survival growth factors and activating key pathways such as PI3K/Akt and FAK, MMP9 may create a microenvironment that facilitates metastatic progression [28]. However, the precise mechanisms underlying its involvement in anoikis sensitivity in RCC still needs further investigation. This study proposes a direction for anoikis-targeted therapy based on anoikis-related genes and experimentally validates MMP9’s involvement in anoikis and its effects on renal cancer cell lines 786 O and Achn. Consequently, our findings offer novel insights into RCC-targeted therapy, suggesting potential innovative strategies for clinical intervention.

## 2. Results

### 2.1. Construction of ARGs and the nomogram

**Figure 1.**
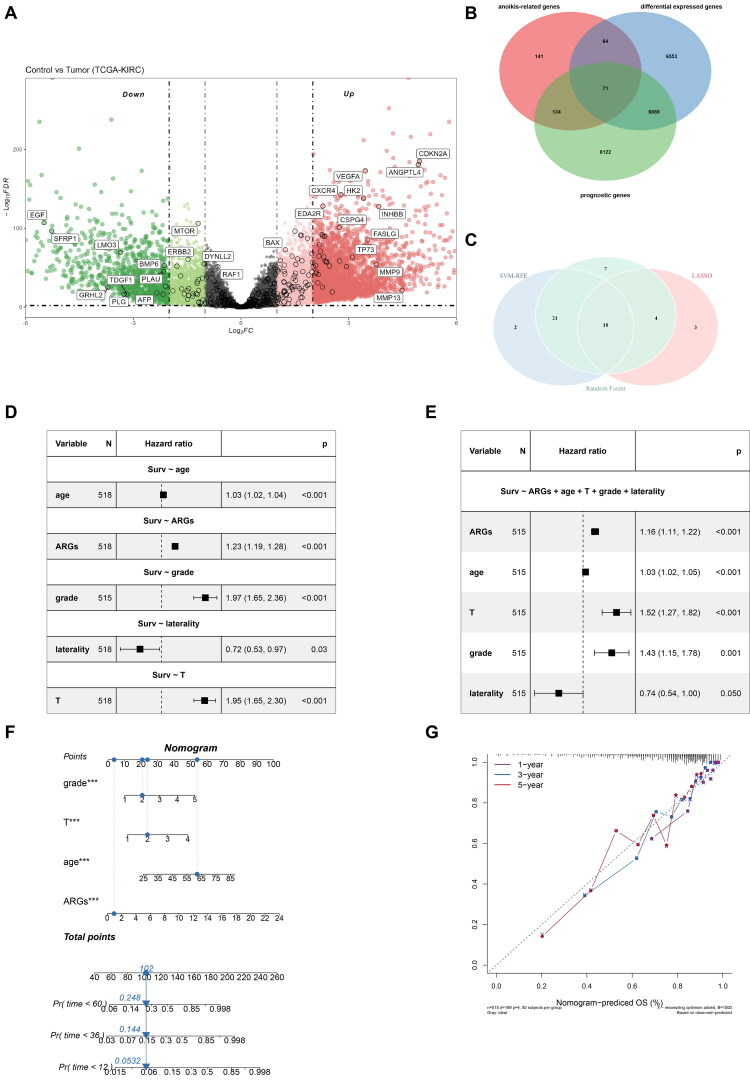
Construction of ARGs and the nomogram. (a) Volcano plot of the DEGs between tumour and normal samples (TCGA-KIRC); (b) Venn plot shows the intersection genes for further screen; (c) Venn plot shows the machine-learning result; (d) Univariate cox regression on ARGs; (e) Multivariate cox regression on ARGs; (f) Nomogram constructed with ARGs and other clinical features; (g) The calibration curves of nomogram.

We aimed to identify ARG associated with ccRCC. In TCGA-KIRC cohort, we took the intersection of DEGs between tumour and normal samples (A total of 9870 upregulated genes and 2906 downregulated genes were identified, Figure 1a), anoikis-related genes (410 genes from GeneCards database) and prognostic genes (14,415 genes with prognosis), resulting in 71 genes for further screening (Figure 1b). The LASSO, SVM-RFE and RF algorithm were conducted with these genes separately. In the LASSO cox regression, 25 genes were selected based on the optimal value of λ (Supplementary Figure 1A, 1B). The SVM-RFE algorithm predicted the most accurate results with 41 genes (Supplementary Figure 1C). For the RF algorithm, we shortlisted the top 50 genes with significant importance (Supplementary Figure 1D). By taking the intersection of the results of the three machine-learning algorithms, we identified 18 robust signature genes (IRF6, CEACAM4, EDAR, SHC1, HMOX1, PTK6, ADCY10, BAG1, MNX1, KL, PLG, LTB4R2, EDA2R, BID, PLK1, HMCN1, PIK3CG and PLAUR, Figure 1c). We constructed coxph models for each patient (518 from TCGA-KIRC; 101 from E-MTAB-1980; 74 from GSE22541) using these 18 signature genes to calculate ARG signature (ARGs). Univariate cox regression was applied to analyze these signature genes, and all but two genes (HMOX1 and PIK3CG) were found to be potential prognostic biomarkers (Supplementary Figure 1E). We further performed univariate (HR = 1.23, 95% CI = 1.19–1.28, *p* < 0.001) and multivariate (HR = 1.16, 95% CI = 1.11–1.22, *p* < 0.001) cox regression on ARGs with clinical features. The results indicated that ARGs is an independent prognostic factor significantly associated with worse overall survival (OS) time in ccRCC (Figure 1d, Figure 1e). A nomogram was constructed with ARGs, grade, T stage and age to enhance the clinical application of ARGs (Figure 1f). The calibration curves of 1-, 3- and 5-year demonstrated that the nomogram had a relatively good predictive performance (Figure 1g).

### 2.2. The prognostic value of ARGs

**Figure 2.**
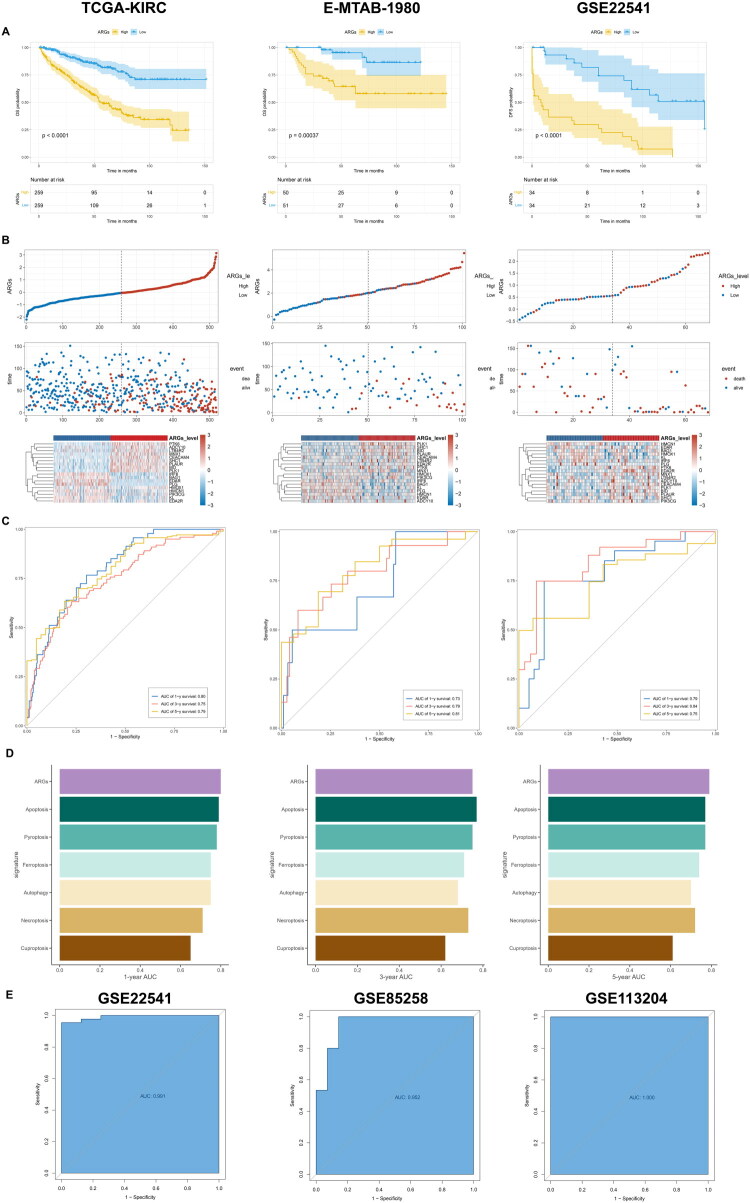
The prognostic value of ARGs. (a) K-M plots of two groups in three datasets; (b) The distribution of patients and heatmap of DEGs between two groups; (c) Time-ROC curve of ARGs; (d) The AUC of ARGs compared to other published signatures; (e) ROC curves show the ARGs’ efficacy in discriminating primary and metastases ccRCC.

To evaluate and validate the prognostic value of ARGs, we utilized three datasets: TCGA-KIRC as the training cohort, E-MATB-1980 and GSE22541 as the validation cohorts. For each patient, the formula was as follows: $\text{risk score}=\sum_{i=1}^{n}\left({\text{Coef}}_{i}*x_{i}\right),i$ represents each selected signature gene and $x_{i}$ is the relative expression. Patients were divided into ARGs High- and Low-risk group according to the median value of risk score.We initially analyzed the distribution of several clinical features between the two groups of patients in the TCGA-KIRC cohort and found that patients in the ARGs-High group had more advanced grades, T-stages and M-stages (Supplementary Figure 2). Figure 2a illustrated that the ARGs-High group had worse OS in the TCGA-KIRC and E-MATB-1980 cohorts, and worse DFS in the GSE22541 cohort. In these datasets, patients with higher ARGs had a higher case fatality rate compared to patients in the ARGs-Low group. Moreover, the heatmaps depicted the relative expression of signature genes (Figure 2b). Time-ROC curves demonstrated that ARGs had excellent predictive performance: AUC = 0.80 for 1-year, 0.75 for 3-year, 0.79 for 5-year survival rate in TCGA-KIRC, AUC = 0.73 for 1-year, 0.79 for 3-year, 0.81 for 5-year survival rate in E-MATB-1980, AUC = 0.79 for 1-year, 0.84 for 3-year, 0.75 for 5-year survival rate in GSE22541 (Figure 2c). Furthermore, we compared the AUC of ARGs in TCGA-KIRC with other recently published cell death-related signatures, and ARGs had the highest AUC of 1- and 5-year survival (Figure 2d).

In three datasets that included patients with metastatic ccRCC (GSE22541, GSE85258 and GSE113204), we demonstrated that these 18 ARGs genes had excellent efficacy in discriminating primary ccRCC from metastases with AUC = 0.991, 0.952 and 1.000, respectively (Figure 2e).

### 2.3. ARGs predict survival and response to immunotherapy and TKIs

**Figure 3.**
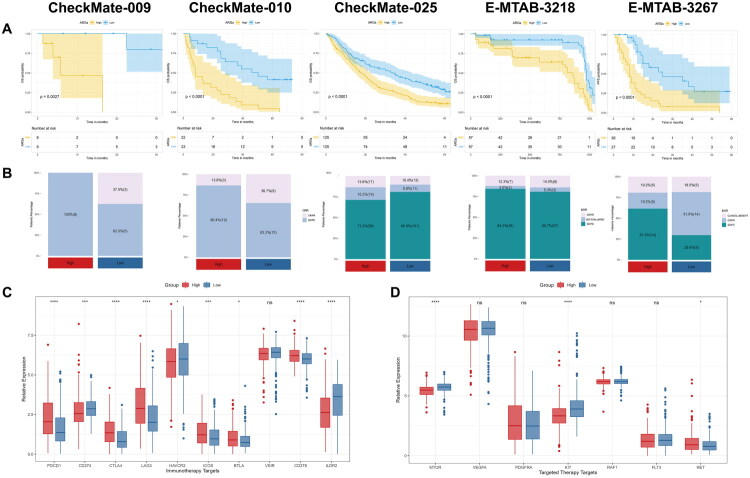
ARGs predict survival and response to immunotherapy and TKIs. (a) K-M plots of two groups in immunotherapy and TKIs treated cohorts; (b) The CR/PR ratio between two groups; (c) Boxplot of immunotherapeutic targets mRNA expression between two groups; (d) Boxplot of targeted-therapeutic targets mRNA expression between two groups.

To evaluate the effectiveness of ARGs as a tool to predict immunotherapy and TKIs sensitivity in ccRCC patients, we analyzed five datasets that included ccRCC patients treated with immunotherapy or TKIs: CheckMate-009 (nivolumab), CheckMate-010 (nivolumab), CheckMate-025 (nivolumab and everolimus), E-MTAB-3218 (nivolumab) and E-MTAB-3267 (sunitinib). Patients were stratified into ARGs-High and ARGs-Low groups as previously described. Strikingly, we found that ARGs still accurately predicted OS or PFS time for patients who received immunotherapy or TKIs (Figure 3a). In four of the five datasets, except CheckMate-025, the proportion of patients who responded to immunotherapy/TKIs (CR/PR) was lower in the ARGs-High group than in the ARGs-Low group (Figure 3b). In addition, we identified significant differential expression of many immunotherapeutic targets and targeted-therapeutic targets between the two groups (Figure 3c, 3d). This result partly explained the distinct response to immunotherapy/TKIs between the ARGs-High and ARGs-Low groups of ccRCC patients.

### 2.4. The potential mechanisms and pathways of ARGs

**Figure 4.**
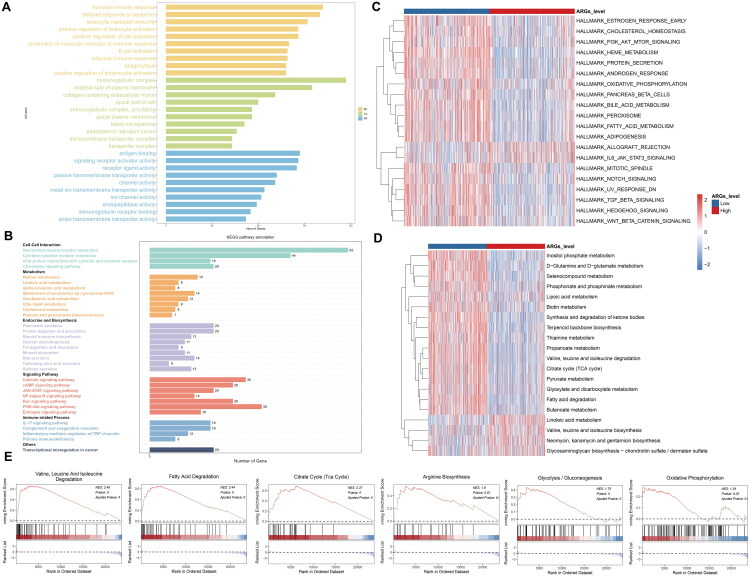
The potential mechanisms and pathways of ARGs. (a) Top GO enrichment; (b) Top KEGG pathway enrichment; (c) GSVA analysis of hallmark pathways; (d) GSVA analysis of metabolic pathways; (e) GSEA analysis of several pathways.

Considering the exceptional prognostic value of ARGs, the underlying mechanism needs to be investigated. We first analyzed the DEGs (Supplementary Figure 3) between the ARGs-High group and the ARGs-Low group by GO/KEGG databases (Figure 4a, 4b). Several biological functions related to tumour growth and anoikis were enriched, including plasma membrane, extracellular matrix and various signalling pathways. In addition, we observed enrichment of several immune-related and metabolic pathways, such as humoral immune response, cytokine-cytokine receptor interaction and retinol metabolism. To compare the activity of these pathways between the two groups, we conducted GSVA analyses in hallmark and metabolic pathways, which revealed differential activation of numerous pathways in these two groups (Figure 4c, 4D). Furthermore, GSEA confirmed that the ARGs-High group exhibited greater activation of valine, leucine and isoleucine degradation, fatty acid degradation, citrate cycle, arginine biosynthesis, glycolysis and oxidative phosphorylation, compared to the ARGs-Low group (Figure 4e). These results suggest that patients with different ARGs may exhibit varying tumour immune microenvironment (TIME) profiles, activation of distinct tumour hallmark pathways and metabolic reprogramming, which ultimately impact prognosis.

### 2.5. ARGs associates with tumour immune microenvironment

**Figure 5.**
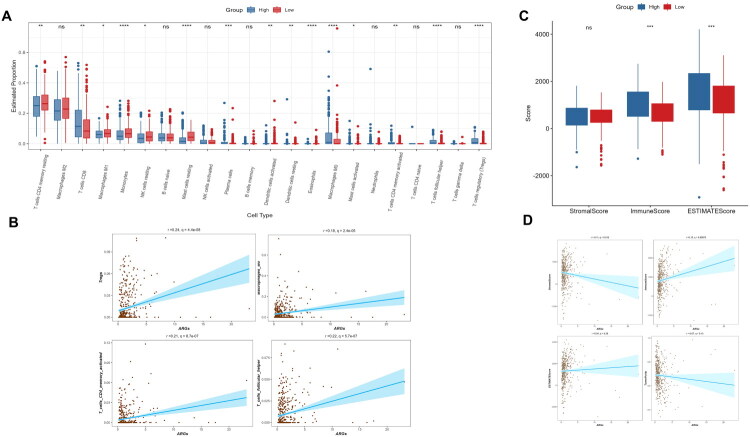
ARGs associates with tumour immune microenvironment. (a) The proportion of immune cells between two groups calculated by CIBERSORT; (b) The correlation between immune cells and ARGs; (c) The immune score calculated by ESTIMATE; (d) The correlation between tumour purity and ARGs.

Further elucidation is necessary to understand the distinct TIME between the two groups and the relationship between ARGs and immune status in ccRCC. Utilizing the CIBERSORT algorithm, we observed a significant reduction in CD4 T memory resting cells, macrophages M1, monocytes, NK resting cells and mast resting cells in the ARGs-High group (Figure 5a). Furthermore, several immune cells exhibited a positive correlation with ARGs, including macrophages M0, CD4 T memory activated cells, T follicular helper cells and Tregs (Figure 5b). NK cells, macrophages and monocytes play crucial roles in anti-tumour immunity [29], while Tregs represent an immunosuppressed state [30], which may explain the low response rate to immunotherapy in the ARGs-High group. However, the immune score calculated using the ESTIMATE algorithm indicated a higher total immune content in the ARGs-High group (Figure 5c), and there was no significant correlation between tumour purity and ARGs (Figure 5d). Therefore, we concluded that ARGs are associated with TIME, but do not significantly affect the proportion of tumour cells.

### 2.6. Somatic mutations atlas of the ARGs high- and low-risk groups

**Figure 6.**
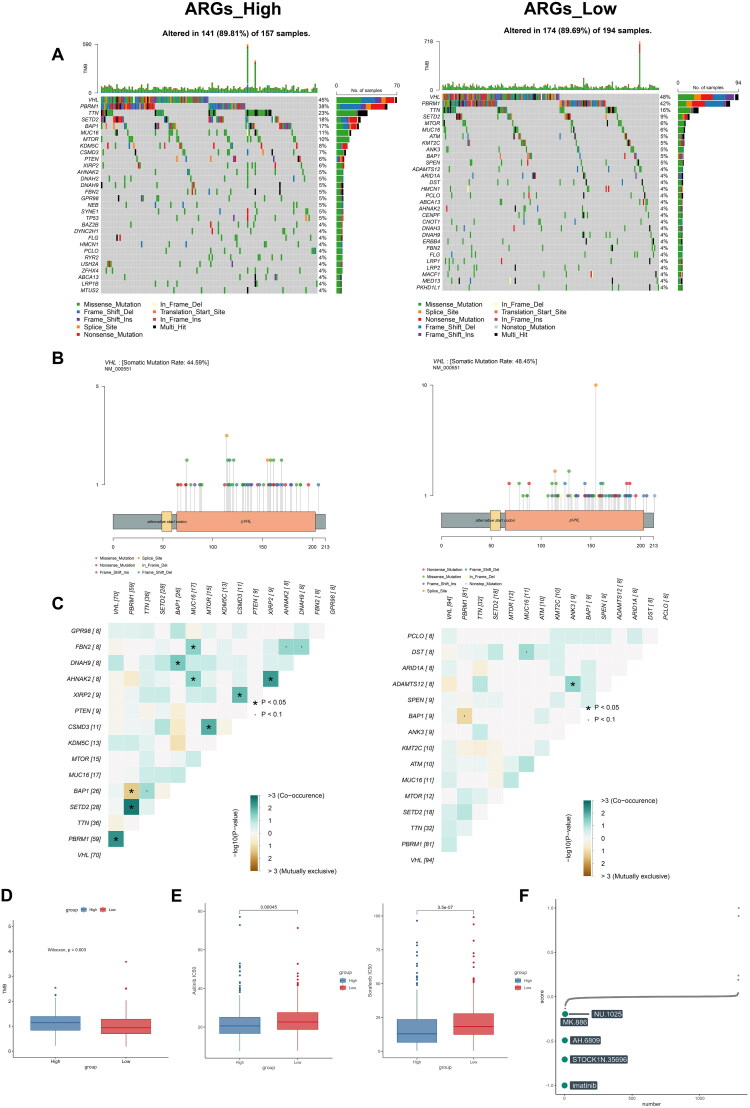
Somatic mutations atlas of the ARGs high- and low-risk groups and candidate drugs. (a) The top mutated genes in two groups; (b) Lolipop plots show the mutation sites of genes; (c) The co-mutations observed by heatmap; (d) Boxplots shows TMB; (e) Estimation of drug sensitivity according to CTRP database; (f) Top 5 candidate compounds of ARGs-high group.

Patients in different ARGs subgroups may exhibit different mutation atlases. We summarized the mutation profiles of the two groups in Supplementary Figure 4, and the top 30 mutated genes in both groups were exhibited in Figure 6a, with little difference observed. The top 3 most frequently mutated genes in both groups were VHL, PBRM1 and TTN, and the lollipop plots indicated different mutation sites of VHL for the two groups (Figure 6b). Intriguingly, a higher likelihood of VHL and PBRM1 co-mutations in the ARGs-High group was observed, while this was not the case in the ARGs-Low group (Figure 6c). Further studies are required to investigate this observation. Notably, the tumour mutational burden (TMB) was statistically higher in the ARGs-High group than in the ARGs-Low group (Figure 6d).

### 2.7. Candidate drug and sensitivity prediction

Axitinib and Sorafenib are both clinically used in the treatment of ccRCC patients. Predictions made using the CTRP database revealed that patients in the ARGs-High group were more sensitive to these two drugs, potentially guiding clinical use (Figure 6e). Additionally, we predicted potential drugs targeting ARGs *via* the CMap_XSum algorithm, and the top 5 candidate compounds were imatinib, STOCK1N.35696, AH.6809, MK.886 and NU.1025 (Figure 6f).

### 2.8. Cell annotations and scoring of scRNA-seq data

**Figure 7.**
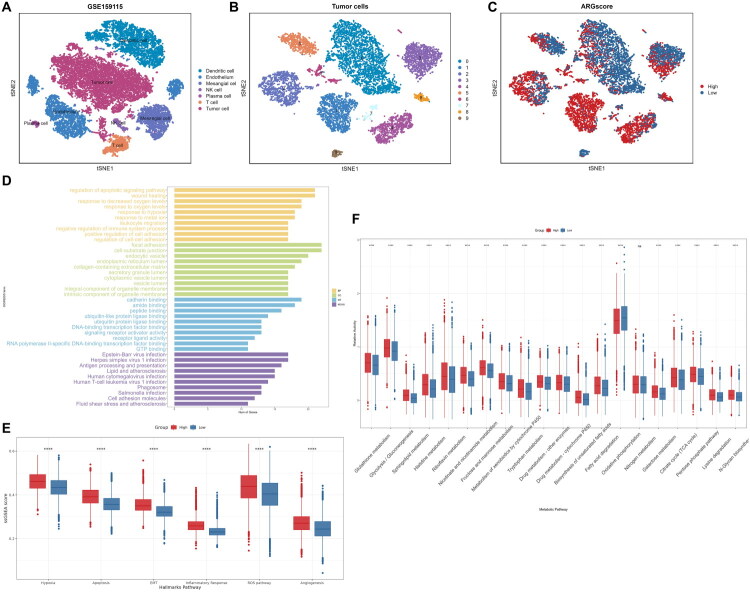
Heterogeneity between the two groups of tumour cells at single-cell level. (a) tSNE plot shows all cells in GSE159115; (b) tSNE plot shows tumour cells with different Seurat clusters; (c) ARGscore calculated by AddModuleScore; (d) GO/KEGG enrichment between two subgroups of tumour cells; (e) Cancer hallmark pathways between two subgroups; (f) Metabolic pathways between two subgroups.

We integrated the scRNA-seq data of seven ccRCC samples after quality control (Supplementary Figure 5A, 5B). The elbowplot indicated that the finest component numbers were 19 (Supplementary Figure 5C). A total of 20,817 cells were clustered into 16 clusters (resolution = 0.5) and the Uniform Manifold Approximation and Projection (UMAP) plot showed that the batch effect had been removed (Supplementary Figure 5D). The cell type-specific markers we used for cell annotation were as follows: T cell (CD3D); NK cell (KLRD1 and NKG7); plasma cell (IGKC and JCHAIN); Mast cell (KIT and TPSB2); tumour cell (CA9, NDUFA4L2 and SLC17A3); endothelium (PECAM1, PTPRB and KDR); mesangial cell (ACTA2 and PDGFRB) [31] (Supplementary Figure 5E, 5F, 5G). Finally, we identified seven distinct cell types: 9402 tumour cells, 4105 dendritic cells, 3655 endothelium mesangial cells, 949 T cells, 332 NK cells and 186 plasma cells (Figure 7a). We next extracted the tumour cells for further analysis. Similar to the previous procedures, after removed a doublet, a total of 9401 singlet tumour cells were clustered into 10 clusters with a resolution of 0.2 (Figure 7b). We used the ‘AddModuleScore’ function to calculate ARGscore for each tumour cells and divided them into two groups according to the median value of ARGscore (Figure 7c).

### 2.9. Heterogeneity between the two groups of tumour cells

Tumour cells in the two groups may have different biological behaviours and hallmarks, which are related to their malignancy. We conducted GO/KEGG enrichment analysis on the DEGs between the two subgroups of tumour cells. The result shown that many pathways associated with tumour growth and metastasis were enriched, such as regulation of apoptotic signalling pathway, response to hypoxia and cell adhesion molecules (Figure 7d). The boxplot of ssGSEA compared the relative activity of some interested cancer hallmark pathways (for example, hypoxia, epithelial-mesenchymal transition (EMT) and Angiogenesis) between the two subgroups (Figure 7e). The ssGSEA score of apoptosis, hypoxia, EMT, inflammatory response, ROS pathway and angiogenesis were higher in the ARGscore_High group. We also found that many metabolic pathways were upregulated in the ARGscore_High group (Figure 7f). These results suggested that there is heterogeneity among these two groups of tumour cells.

### 2.10. Pseudotime analysis indicates that anoikis resistance may occur as the tumour cell growth

**Figure 8.**
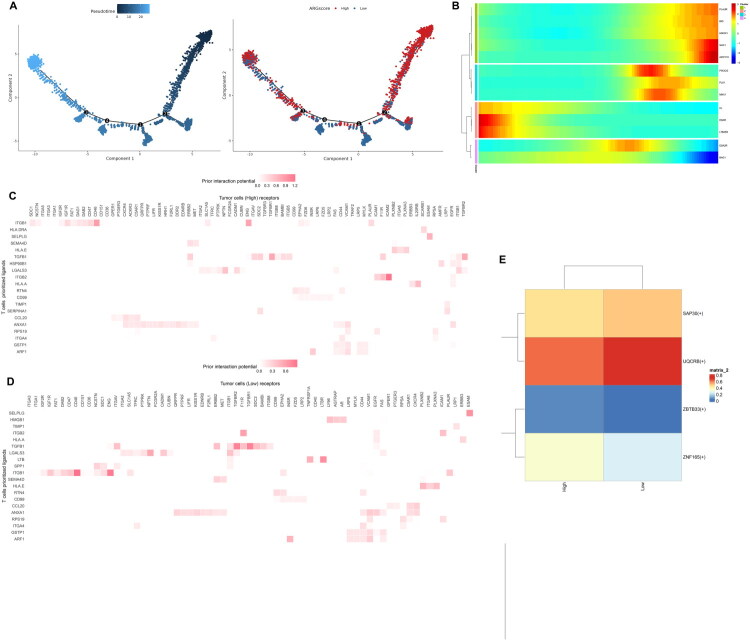
Pseudotime analysis and cell-cell communications. (a) The ARGscores increase with pseudotime; (b) The dynamic change of signature genes during the development of tumour cells; (c) Ligands-receptors-targets regulatory network between ARGs_High tumour cells and T cells; (d) Ligands-receptors-targets regulatory network between ARGs_Low tumour cells and T cells; (e) Scenic predicts the top transcription factors in two subgroups of tumour cells.

In order to depict the dynamic change of ARGscore during tumour cells development, Monocle 2 was employed to portrait a developmental trajectory. Figure 8a demonstrated that ARGscore decreased as the tumour cell growth. Considering that tumour cells in the ARGscore_High group have higher apoptotic activity (Figure 7f), we hypothesis that during tumour development, anoikis resistance occurs in tumour cells and therefore ARGscore decreased, which represents apoptosis pathway’s activity. In addition, the dynamic change in the expression of signature genes during the development of tumour cells was shown in Figure 8b. PLAUR, BID, HMOX1, SHC1 and ADCY10 expression increases with the development trajectory while EDA2R and BAG1 expression decreases.

### 2.11. Ligands-receptors-targets regulatory network and transcription factor analysis in tumour cells

Using NicheNet, we constructed ligands-receptors-targets regulatory networks between T cells and two groups of tumour cells separately. Between ARGs_High tumour cells and T cells, ITGB2-ICAM1 and TGFB1-TGFBR1 were ligand-receptor pairs with strong regulatory potential (Figure 8c), while EDN1, JUNB, SOCS3, VIM and COL1A2 were top genes target to TGFB1 (Supplementary Figure 6A). Between ARGs_Low tumour cells and T cells, ITGB1-CD46 and ITGB1-ENG were ligand-receptor pairs with strong regulatory potential (Figure 8d), while COL1A2, CCL3, SERPINE1, FN1 and CDKN1A were top genes target to TGFB1 (Supplementary Figure 6B). SCENIC predicted the top 4 transcription factors that differed expressed most between the two subgroups of tumour cells: SAP30, UQCRB, ZBTB33 and ZNF165 (Figure 8e).

### 2.12. Establishment of anoikis model and determination of target gene

**Figure 9.**
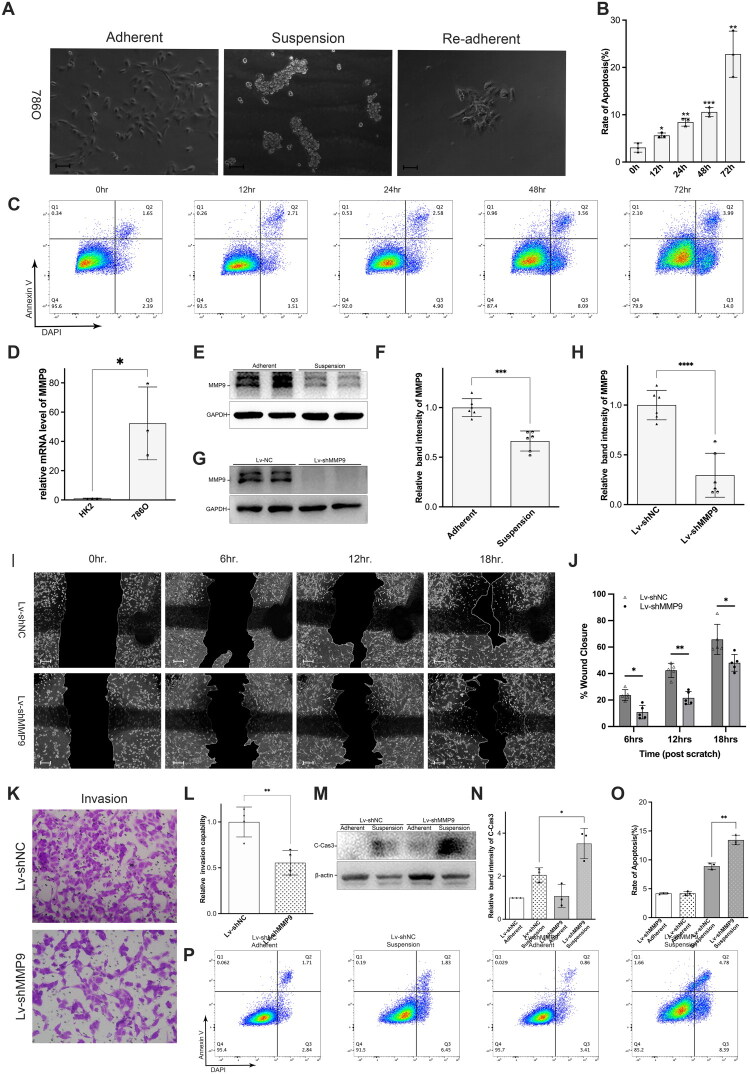
MMP9 was involved in the anoikis resistance of RCC and knockdown of MMP9 significantly attenuated migration by accelerating anoikis in 786 O cells. (a) Morphological representation of anoikis models established by poly-HEMA, scale bars: 50 μm; (b & c) Flowcytometry analysis showed that the percentage of apoptosis increased with the duration of poly-HEMA treatment; (d) qRT-PCR analysis showed significant overexpression of MMP9 in 786 O cell line compared to HK-2 cell line; (e & f) Western blot studies showed that MMP9 was downregulated in poly-HEMA-treated 786 O cells; (g & h) Western blot studies confirmed lentivirus-mediated knockdown of MMP9; (i & j) Wound-healing assay indicated knockdown of MMP9 attenuated migration capacity of 786 O cells, scale bars: 100 μm; (k & l). Transwell assay indicated knockdown of MMP9 alleviated invasion capacity of 786 O cells; (m – p) Flow cytometry analysis and western blot studies demonstrated that knockdown of MMP9 significantly aggravated poly-HEMA induced anoikis. *p < 0.05, **p < 0.01, ***p < 0.001, ****p < 0.0001.

In this study, we investigated the invasion and metastasis of renal clear cell carcinoma using an anoikis model. Following the protocol outlined by Valentinis et al. [32], we employed poly-HEMA-coated Petri dishes to maintain cells in suspension, thereby inducing anoikis, an anchorage-dependent form of programmed cell death. Cells in the suspension group formed clusters and were unable to adhere to the dish, as opposed to the control group (Figure 9b). Interestingly, after 72 h in suspension, cells began to reattach, albeit at a slow rate, when transferred to normal Petri dishes for 24 h (Figure 9b), suggesting partial reactivation. To determine the optimal time for establishing the anoikis model, 786 O cells were treated with poly-HEMA for 0, 12, 24, 48 and 72 h and analyzed by flow cytometry. Our findings demonstrated a gradual increase in anoikis rates as treatment duration increased, with significant damage observed at 72 h (Figure 9g, 9h). Based on the moderate damage observed at 48 h, we selected this time point for all subsequent experiments.

Although the ARGs prognostic model we previously constructed has shown good prognostic predictive performance, this does not mean that all the genes within the model possess strong functions, such as the majority of pseudogenes [33]. In order to explore potential clinical translational targets of anoikis in ccRCC, it is necessary to re-screen the genes that might have robust functions from scratch. Anoikis is related to tumour metastasis. In order to further screen the key anoikis genes in the process of ccRCC metastasis, we intersected anoikis-related genes, DEGs from TCGA-KIRC, and DEGs from GSE22541 (ccRCC with metastasis) and finally obtained five candidate genes: MMP9, BIRC3, MUC1, HMCN1 and ANGPTL4 (Supplementary Figure 7A). Of these, only MMP9 was associated with worse OS and RFS (Supplementary Figure 7B, C). MMP9 mRNA expression was elevated in ccRCC patient samples and was higher in metastatic ccRCC than non-metastatic (Supplementary Figure 7D, E). Futhermore, MMP9 mRNA expression was significantly elevated in ARGs_high group (Supplementary Figure 7F). RT-qPCR analysis validated that MMP9 expression was significantly upregulated in 786 O cells (human renal clear cell carcinoma) compared to HK-2 cells (human renal cortical proximal convoluted tubule cell) (Figure 9a). Additionally, western blot analysis confirmed that MMP9 was markedly downregulated in 786 O cells treated with poly-HEMA for 48 h (Figure 9c, 9e), suggesting an important role for MMP9 in anoikis of renal clear cell carcinoma.

### 2.13. MMP9 knockdown significantly inhibits 786 O cell migration by exacerbating anoikis

To further investigate the relationship between MMP9 and anoikis, we employed a lentivirus to suppress MMP9 expression in 786 O cells. Western blot analysis validated the reduction in MMP9 expression (Figure 9g, 9h). The wound-healing assay demonstrated that MMP9 knockdown significantly reduced the migration rate of 786 O cells for about 30% compared to the shNC group (Figure 9i, 9j). These findings were further corroborated by transwell assay results, which confirmed that MMP9 knockdown revealed a marked decrease in the invasive capacity of 786 O cells for about 50% (Figure 9k, 9l). Together, these experiments indicate that MMP9 is involved in the invasion and metastasis of ccRCC and that downregulation of MMP9 expression attenuates this process. Moreover, cleaved caspase 3, an apoptosis-related marker, was upregulated in MMP9-knockdown 786 O cells treated with poly-HEMA, as shown by western blot analysis (Figure 9m, 9n). Similarly, flow cytometry data confirmed that MMP9 downregulation substantially increased the apoptosis rate of 786 O cells for about 59% following poly-HEMA treatment (Figure 9o, 9p).

### 2.14. Similar results were observed in another renal carcinoma cell line Achn

**Figure 10.**
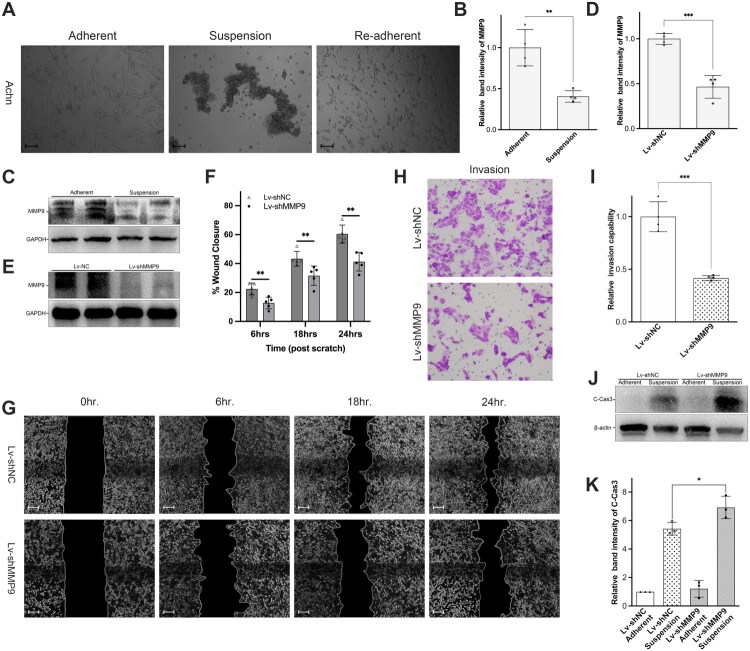
MMP9 knockdown attenuated migration by exacerbating anoikis in Achn cells as well. (a) Morphological representation of Achn anoikis models established by poly-HEMA, scale bars: 50 μm; (b & c) Western blot studies showed that MMP9 was downregulated in poly-HEMA-treated Achn cells; (d & e) Western blot studies confirmed lentivirus-mediated knockdown of MMP9; (f & g) Wound-healing assay indicated knockdown of MMP9 attenuated migration of Achn cells, scale bars: 100 μm; (h & i). Transwell assay alleviated invasion capacity of Achn cells; (j & k). Western blot studies demonstrated that knockdown of MMP9 significantly aggravated poly-HEMA induced anoikis in Achn cells. *p < 0.05, **p < 0.01, ***p < 0.001.

While the efficacy of regulating anoikis to reduce invasion and metastasis of ccRCC was demonstrated in 786 O cell lines, solid tumours and cell lines are known for their heterogeneity. To more comprehensively assess the potential value of regulating anoikis in RCC, we employed another renal carcinoma cell line, Achn, to validate our findings, and observed similar results. Achn cells formed clusters in poly-HEMA-coated Petri dishes but reattached and partially regained their original morphology when transferred to normal Petri dishes (Figure 10a), consistent with our observations in 786 O cells. Moreover, MMP9 expression was also downregulated in poly-HEMA-treated Achn cells (Figure 10b, 10c). To further investigate the underlying mechanism, we established an MMP9-knockdown Achn cell line using lentivirus, as previously performed, and confirmed the knockdown by western blot analysis (Figure 10d, 10e). We then conducted a wound-healing assay in both the shMMP9 and shNC groups. Despite adjusting the observation time points to 6, 18 and 24 h due to the differing growth rates of Achn and 786 O cells, the results remained consistent (Figure 10f, 10g). The shMMP9 group demonstrated significantly slower wound healing compared to the shNC group. Furthermore, transwell assays performed in the Achn cell line mirrored the findings observed in 786 O cells, with MMP9 knockdown markedly reducing cellular invasion (Figure 10h, 10i). These results collectively suggest that MMP9 downregulation effectively mitigates invasion and metastasis across multiple RCC cell lines. Western blot analysis of cleaved caspase 3 also indicated that decreased MMP9 notably augmented apoptosis following poly-HEMA treatment (Figure 10j, 10k). Altogether, our data reveal that downregulating MMP9 expression in poly-HEMA-treated 786 O and Achn cells substantially accelerates the anoikis process, consequently inhibiting their migration and metastasis potential.

## 3. Discussions

In this study, we constructed a novel ARGs prognostic model that effectively stratifies ccRCC patients into two ARG-based risk groups using various machine-learning algorithms. To ensure the robustness of our prognostic model, we applied LASSO, SVM-RFE and Random Forest algorithms, which are widely used for high-dimensional data analysis. These methods complement each other: LASSO effectively selects key variables by imposing regularization, SVM-RFE refines feature selection in complex data structures, and Random Forest provides stable feature ranking. By integrating these approaches, we aimed to enhance the reliability of our selected gene signature.

Our analysis identified 18 dependable ARGs that play key roles in predicting prognosis and therapeutic responses in ccRCC. These genes highlight the intricate balance between cell survival, adhesion and extracellular remodelling, which are fundamental processes in tumour progression. Notably, genes such as SHC1 and PTK6 regulate essential signal transduction pathways, including the MAPK and PI3K cascades, which are critical for both tumour cell proliferation and resistance to programmed cell death—mechanisms that have been strongly implicated in therapeutic resistance in ccRCC [34,35]. Meanwhile, regulators of apoptosis like BID and BAG1 may promote anoikis resistance, facilitating metastatic progression, a phenomenon previously described in RCC and other aggressive cancers [35,36]. Additionally, genes involved in extracellular matrix remodelling and adhesion, such as PLAUR, PLG and CEACAM4, may enhance tumour invasion by modifying the tumour microenvironment, a process extensively reported in invasive malignancies [37–39]. Interestingly, IRF6, EDAR and EDA2R, which regulate epithelial differentiation, may further contribute to ccRCC progression by influencing cellular plasticity [40,41]. Other genes such as KL, MNX1, HMCN1 and ADCY10 suggest that anoikis resistance is not restricted to survival pathways but also extends to metabolic and proliferative mechanisms that drive both tumour aggressiveness and treatment resistance [42–45]. At the single-cell level, our analyses revealed distinct ccRCC subpopulations with varying levels of ARG expression, underscoring tumour heterogeneity. Developmental trajectory analysis further suggests that anoikis resistance may emerge as tumours evolve, potentially driving increased metastatic capacity. In addition, we identified a ligand-receptor-target regulatory network and key transcription factors involved in anoikis resistance, providing mechanistic insights that may guide future therapeutic interventions.

Building on these molecular insights, we evaluated the prognostic significance of the ARGs across multiple independent datasets. Our results indicate that this ARG-based risk model is a stable independent prognostic factor for ccRCC, with significant associations with OS in both univariate (HR = 1.23, 95% CI: 1.19–1.28) and multivariate (HR = 1.16, 95% CI: 1.11–1.22) analyses. These HR values reinforce the clinical relevance of ARG expression, as higher risk scores consistently correlated with poorer patient outcomes. Notably, compared with previously published ARG-based prognostic models for RCC, which reported HR values ranging from 1.017 to 1.215 [46–49], our model falls within a similar or slightly superior range, further supporting its reliability. In addition, our ARGs achieved a 5-year AUC of 0.79, notably outperforming previously reported models (AUC range: 0.671–0.736) [46–52]. This enhanced predictive accuracy underscores the clinical utility of our model in risk stratification and treatment decision-making. Moreover, our comprehensive validation across three independent cohorts (TCGA-KIRC, *n* = 518; E-MTAB-1980, *n* = 101; GSE22541, *n* = 74) ensures greater generalizability compared to previous models, which often relied on smaller or single-cohort datasets [46–52]. Despite minor variations in HR values across datasets, the overall trend remained consistent, demonstrating that high-risk ARG expression is robustly associated with poor survival. Such multi-cohort validation reinforces the clinical relevance and translational potential of our findings, supporting the integration of ARGs into prognostic models for personalized RCC management.

The reliable performance of our ARGs across diverse datasets has direct implications for clinical management strategies in RCC. Given that various molecular signatures related to tumour biology, including those involved in tumour immune and tumour microenvironment interactions, have been demonstrated to predict differential treatment responses in RCC [53–55], our ARGs represents a promising predictive tool for personalized therapy selection. Particularly, this signature may help stratify patients who would benefit most from immune checkpoint inhibitors or TKIs, both standard treatments for advanced RCC with variable response rates [7]. The stable and reproducible nature of our ARGs allows clinicians to potentially select optimal therapeutic combinations based on individual risk profiles derived from our model. Furthermore, the identification of specific ARGs involved in treatment resistance mechanisms provides promising avenues for developing combination therapies that could integrate conventional treatments with novel agents targeting ARG-driven pathways, potentially overcoming resistance mechanisms and improving patient outcomes.

While our primary prognostic model is based on the 18 ARGs associated with overall survival, our metastatic subgroup analysis identified MMP9 as a critical molecular bridge linking anoikis resistance to the metastatic cascade in ccRCC. MMP9 functions as an important mediator of processes essential for tumour development, including ECM remodelling, EMT, cell migration, angiogenesis and immune response modulation [24]. Particularly relevant to anoikis resistance, MMP9 regulates EMT through the PI3K/AKT/NF-κB signalling pathway—a critical connection since EMT has been established as not only essential for tumour invasion but also a key feature in anoikis resistance development [56–58]. Additionally, as a primary enzyme in ECM degradation, MMP9 works in concert with molecules such as SMAD2/3 and TIM3 [59–61], further reinforcing its role in anoikis regulation. Our experimental findings support these mechanistic connections: MMP9 expression was significantly elevated in the RCC cell line 786 O compared to normal kidney HK-2 cells (Figure 9a), suggesting its importance in RCC pathogenesis. Moreover, in our poly-HEMA-induced extracellular matrix deprivation model, which simulates conditions promoting anoikis, MMP9 expression was significantly reduced (Figure 9c and 9e), providing potential evidence of its involvement in anoikis-related pathways in RCC.

Anoikis resistance represents a fundamental mechanism in tumour metastasis, enabling cancer cells to survive despite detachment from the extracellular matrix. Previous research has identified several factors regulating anoikis sensitivity in RCC, complementing our findings on ARGs and MMP9. Shinichi and Meredith demonstrated that quinazoline-based drugs induce anoikis in RCC by disrupting survival signals in focal adhesions [21,62], while other studies showed that knockdown of HMGA1 and TIM-3 enhances anoikis occurrence [63], and silencing the tumour suppressor DLK1 increases anoikis resistance. These findings align with our observations regarding the complex interplay between adhesion-related ARGs and metastatic potential. Furthermore, recent experimental approaches such as Lu Wang’s ECM deprivation system effectively reversed RCC’s anoikis resistance and enhanced sensitivity to doxorubicin chemotherapy [64], while Peng Zhang’s team found that silencing TrkB improved sorafenib efficacy [65]. These therapeutic strategies highlight an important implication of our ARG analysis: targeting anoikis resistance pathways may not only directly inhibit metastasis but also synergize with existing treatments, creating new therapeutic opportunities for RCC. However, despite these advances, comprehensive models reflecting the prognostic impact of anoikis-related genes have been limited. Recent anoikis-based RCC risk prediction models by Zhuo Chen and Jin Wang represent progress in this direction, though they still require robust experimental validation [49,66]—a gap our current ARGs and MMP9’s experimental findings help address.

In this study, we employed flow cytometry to assess apoptosis rates and conducted wound-healing/transwell assays to evaluate the invasive and migratory capabilities of RCC cells. Our results indicated that following MMP9 gene knockdown, the proportion of anoikis cells increased in both the 786 O and Achn cell lines, while their invasive and metastatic capacities decreased. This suggests that MMP9 functions as an inhibitor of anoikis in RCC (Figures 9 and 10). Notably, this study is the first to demonstrate MMP9’s regulatory role in anoikis within the context of RCC. While previous research has established MMP9’s involvement in various mechanisms of tumour invasion and metastasis, it is essential to recognize that these capabilities do not necessarily encompass or equate to anoikis resistance. Therefore, further experiments are warranted to elucidate the specific molecular pathways through which MMP9 regulates anoikis in RCC.

Despite the insights gained from our research, several limitations should be acknowledged. First, while our study leverages bioinformatics analysis alongside clinical specimens, potential biases in data selection could influence the robustness of our findings. Sample size limitations may restrict the generalizability of our results, as larger cohorts are needed to validate the prognostic significance of MMP9 and other ARGs across diverse patient populations. Additionally, confounding factors, such as variations in tumour microenvironments and patient treatment histories, may affect the interpretation of our results. From a translational perspective, MMP9 inhibitors or siRNA-based therapies offer promising avenues for enhancing anoikis sensitivity in RCC. Novel drug delivery systems, such as nanoparticles, could facilitate targeted delivery of these therapeutics, potentially maximizing efficacy while minimizing systemic side effects.

## 4. Conclusions

In conclusion, this study presents a novel approach to understanding RCC through the identification and analysis of ARGs, with a particular focus on MMP9. By combining bioinformatics and experimental validation, we provide compelling evidence that MMP9 plays a critical role in regulating Anoikis resistance, a key factor in RCC progression and metastasis. Our findings suggest that targeting MMP9 could offer a promising therapeutic strategy for overcoming Anoikis resistance, potentially improving the efficacy of existing treatments such as immune checkpoint inhibitors and TKIs. Furthermore, the identification of ARGs in this study lays the foundation for developing personalized treatment strategies that not only predict prognosis but also help tailor therapies based on specific molecular profiles. As we continue to explore the relationship between ARGs and RCC, this research may contribute to advancing more effective, targeted therapies for RCC patients, ultimately improving clinical outcomes and quality of life. By addressing Anoikis resistance, we may open new avenues for cancer treatment, not only for RCC but also for other metastatic cancers.

## 5. Methods

### 5.1. Data acquisition

Table 1 provides a brief summary of the datasets used in this manuscript. The data for this study were collected in October 2023.

**Table 1.** Summary of datasets used in current study.

Datasets	Brief description (*n* = enrolled)	Type
TCGA^a^-KIRC^b^	ccRCC^c^ patients (*n* = 518) and healthy controls (*n* = 71)	RNA-seq^d^
E-MTAB-1980	ccRCC patients (*n* = 101)	RNA-seq
E-MTAB-3218	ccRCC patients (*n* = 114) treated by nivolumab	RNA-seq
E-MTAB-3267	ccRCC patients (*n* = 59) treated by sunitinib	RNA-seq
CHECKMATE-009	ccRCC patients (*n* = 16) treated by nivolumab	RNA-seq
CHECKMATE-010	ccRCC patients (*n* = 45) treated by nivolumab	RNA-seq
CHECKMATE-025	ccRCC patients (*n* = 250) treated by nivolumab and everolimus	RNA-seq
GSE22541	Primary and pulmonary metastasis of ccRCC (*n* = 68)	RNA-seq
GSE85258	Primary and pulmonary metastasis of ccRCC (*n* = 29)	RNA-seq
GSE113204	Primary, pulmonary metastasis and tumour thrombus of ccRCC (*n* = 14)	RNA-seq
GSE159115	ccRCC patients (*n* = 7)	scRNA-seq^e^

^a^TCGA: The Cancer Genome Atlas.

^b^KIRC: Kidney Renal Clear Cell Carcinoma.

^c^ccRCC: Clear cell renal cell carcinoma.

^d^RNA-seq: RNA sequencing.

^e^scRNA-seq: Single-cell RNA sequencing.

We downloaded the RNA-seq counts data and corresponding survival data of The Cancer Genome Atlas of kidney renal clear cell carcinoma (TCGA-KIRC) cohort *via* UCSC Xena (http://xena.ucsc.edu/). Genes expressed in less than half of the patients were excluded, and patients with a survival time of less than 30 days were removed. A total of 518 ccRCC patients, 71 healthy controls and 25,503 genes were enrolled. The somatic mutation data of TCGA-KIRC cohort was obtained from TCGA database by R package ‘TCGAmutations’. The transcription profiling data and survival data of E-MTAB-1980 [67], E-MTAB-3218 [68] and E-MTAB-3267 [69] were download from the ArrayExpress database (https://www.ebi.ac.uk/biostudies/arrayexpress). The RNA-seq data of GSE22541 [70], GSE85258 [71], GSE113204 [72] and the scRNA-seq data of GSE159115 [73] were download from the Gene Expression Omnibus (GEO) database (https://www.ncbi.nlm.nih.gov/geo/). The transcription profiling and survival data of ccRCC patients treated by PD-1 blocker or TKIs were obtained from published randomized trial (CheckMate-009/010/025) [55].

We collected 410 anoikis-related genes from the GeneCards databases (https://www.genecards.org/) with the Relevance Scores > 0.5 (Supplementary Table S1).

### 5.2. Differential expression analysis and machine learning

R package ‘DESeq2’[74] was employed to screened the differentially expressed genes (DEGs) between the control and ccRCC in the TCGA-KIRC cohort according to the following criteria: adjusted *p*-value < 0.05 and | Log_2_(fold-change) | >1. For each gene, we divided the patients into two groups according to the median expression value and performed log-rank test. Genes with *p* < 0.05 were defined as prognostic genes. The intersection of anoikis-related genes, DEGs and prognostic genes were further analyzed by machine learning.

We used three machine-learning algorithms to obtain the best subset of candidate genes. The least absolute shrinkage and selection operator (LASSO) [75] algorithm was performed by R package ‘glmnet’ (alpha = 1). The minimum penalty parameter (λ) was selected by tenfold cross-validation. Support vector machine-recursive feature elimination (SVM-RFE) [76] was used to identify candidate genes with Structural Risk Minimization Principle by R package ‘e1071’ (cost = 1, kernel= ‘radial’). The random forest (RF) [77] algorithm was performed by R package ‘randomForest’(ntree = 500) and chosen the top 50 genes with great importance. Genes used to establish the Anoikis-Related Genes signature (ARGs) were selected by taking the intersection of the result of three algorithms.

### 5.3. ARGs establishment and construction of a nomogram

In each dataset, ARGs was established by cox proportional-hazards model with selected signature genes. For each patient, the formula was as follows: $\text{risk score}=\sum_{i=1}^{n}\left({\text{Coef}}_{i}*x_{i}\right),i$ represents each selected signature gene and $x_{i}$ is the relative expression. Patients were divided into ARGs High- and Low-risk group according to the median value of risk score. patients with a risk score above the median were labelled ‘High-risk’ and those below were ‘Low-risk’.

In TCGA-KIRC cohort, we collected the clinical data of ccRCC patients (age, grade, T stage and laterality). Together with ARGs, univariate and multivariate cox regression analysis was performed on these classical clinical features by R package ‘ezcox’ (https://github.com/ShixiangWang/ezcox) [78]. To facilitate clinical application by urologist, a prognostic nomogram was constructed by R package ‘regplot’. The 1-, 3- and 5-year calibration curves were shown to observe nomogram performance by bootstrapping (1000 times).

### 5.4. Survival analysis

In datasets with survival information, R packages ‘survival’ and ‘survminer’ were used to compare the OS or DFS between the two groups of ccRCC patients by log-rank test and Kaplan-Meier curves were generated. To assess the sensitivity and specificity of ARGs in predicting survival, receiver operating characteristic (ROC) curves were depicted AUC was calculated by R package ‘TimeROC’.

Given that anoikis plays and crucial role in suppressing tumour metastasis, we tested whether these selected signature genes could discriminate primary tumour from metastases and evaluated their performance by ROC curves in three datasets which including primary ccRCC and mccRCC patients: GSE22541, GSE85258 and GSE113204.

Additionally, in TCGA-KIRC cohort, we compared the 1-, 3- and 5-year AUC of our signature to other recently published cell death related gene signatures: a cupoptosis-related gene signature by Ye et al. [79], a pyroptosis-related gene signature by Xu et al. [80], a apoptosis-related gene signature by Huang et al. [81], a necroptosis-related gene signature by Sun et al. [82], a autophagy-related gene signature by Chong et al. [83], a ferroptosis-related gene signature by Zhou et al. [84].

### 5.5. Enrichment analysis

In TCGA-KIRC cohort, we firstly performed differentially expressed genes analysis between the High- and Low-ARGs groups to investigate underlying pathways of ARGs. DEGs were input in Gene Ontology (GO) and Kyoto Encyclopaedia of Genes and Genomes (KEGG) databases using R package ‘clusterProfiler’[85] to perform Over Representation Analysis. The Gene Set Variation Analysis (GSVA) scores of 50 hallmark pathways and metabolism pathways were calculated and compared between the two groups by R package ‘GSVA’. The pathway information files were obtained from the MSigDB database [86,87] and R package ‘scMetabolism’[88]. Gene Set Enrichment Analysis (GSEA) was performed by R package ‘GSEABase’ and visualized by R package ‘GseaVis’ (https://github.com/junjunlab/GseaVis). Pathways with FDR < 0.25, *p* < 0.05 and |NES| > 1 were considered enrich significantly.

### 5.6. Depicting tumour immune microenvironment

To compare the estimated proportion of immune cells in tumour immune microenvironment (TIME) between the two groups of ccRCC in TCGA-KIRC cohort, we utilized the CIBERSORT algorithm [89]. In addition, the ESTIMATE algorithm [90] was used to infer tumour purity and calculate StromalScore, ImmuneScore and ESTIMATEScore. The relationship between ARGs and these TIME features was explored by Spearman’s correlation analysis.

### 5.7. Prediction of potential therapeutic targets and drug sensitivity

Since many drugs may be or have been clinically used to ccRCC patients, it is important to determine if there is a distinct sensitivity of these drugs in the High- and Low-ARGs groups. We downloaded the data on the sensitivity of many compounds to multiple cancer cell lines from the Cancer Therapeutic Response Portal (CTRP) database [91–93] and used R package ‘oncoPredict’[94] to predict the sensitivity of patients in TCGA-KIRC cohort to certain drugs. 50% inhibiting concentration (IC50) was used to represent the drug sensitivity.

ARGs may be a potential therapeutic target. The data gained from CMap database was analyzed by eXtreme Sum algorithm [95] to search for drugs that might target the DEGs between the two groups.

### 5.8. Processing scRNA-seq data

GSE159115 dataset includes the scRNA-seq data (10X Genomics) from seven ccRCC patients and we converted the row count matrix into a Seurat object by R package ‘Seurat’ (version 3.0.0) [96]. Cells with fewer than 200 unique molecular identifier (UMI) counts or with more than 20% mitochondrial-related UMI counts were removed. R package ‘DoubletFinder’[97] was employed to identify and remove potential doublets with a DoubletRate of 7.6% for 10,000 cells. After quality control, we removed the potential batch effect by R package ‘harmony’[98] and obtained a new integrated data matrix. The matrix was log normalized, scaled and centred by ‘NormalizeData’ and ‘ScaleData’ function. In order to reduce the dimensionality, we conducted principal component analysis (PCA) and find the finest component numbers by ‘Elbowplot’ function. We identified the main cell clusters with a resolution of 0.5 and visualized them by ‘FindNeighbors’, ‘FindClusters’, ‘RunTSNE’ and ‘RunUMAP’ function. A list of cell markers from a published manuscript [31] was used to annotate all cells. ‘AddModuleScore’ function was used to calculate module scores of 410 anoikis-related genes (ARGscore) for each tumour cell, and divided all tumour cells into two groups based on the median value of ARGscore. Because the lack of interest in ribosome pathways, we used ‘FindMarkers’ function to find DEGs between the two groups of cells after removing ribosome genes (|Log2(fold-change) | > 0.5 and *p*-adjust < 0.05). GO/KEGG analysis, single sample gene set enrichment analysis (ssGSEA) by R package ‘GSVA’ and metabolic pathway analysis by R package ‘scMetabolism’[88] were performed between the two groups of tumour cells.

### 5.9. Pseudotime analysis

To investigate whether anoikis resistance occurs during ccRCC tumour cells growth, we placed all tumour cells on a trajectory calculated by Monocle2 [99–101] and analyzed the dynamic changes in their ARGscore. ‘plot_pesudotime_heatmap’ function revealed distinct expression patterns of key genes.

### 5.10. Cell-cell interaction analysis

To depict the ligands-receptors-transcriptional regulators-targets networks of the two groups of tumour cells, we employed R package ‘nichenetr’[102]. Considering that T cells are one of the pivotal factors in current immunotherapy [103], we focused on the potential regulatory network between tumour cells and T cells.

### 5.11. Transcription factors analysis

In order to identify preferentially expressed regulons between the two groups of tumour cells, pySCENIC [104] was performed based on python. ‘calcRSS’ function was used to calculate the regulon specificity score of each group and ΔFoldChange represents the expression difference.

### 5.12. Cell culture

Our experiments were conducted using HK-2 cells (RRID: CVCL_0302), 786 O cells (RRID: CVCL_1051) and Achn cells (RRID: CVCL_1067). All cell lines were obtained from Abiowell Biotechnology Co., Ltd., authenticated by STR profiling within the past 3 years, and tested for mycoplasma contamination. The 786 O cells were cultured in RPMI-1640 medium (supplemented with 10% FBS and 1% antibiotics), MEM medium for Achn cells, and low glucose DMEM for HK-2 cells. All cells were maintained at 37 °C in a 5% CO2 atmosphere. Lentivirus Lv-shMMP9 was employed to knockdown MMP9 expression in 786 O and Achn cells, with Lv-shNC serving as a control. Stably transfected cell lines were selected using puromycin (3 μg/ml, 48 h).

An anoikis model was established by culturing cells on poly-HEMA (Sigma)-coated Petri dishes for 12–72 h, as previously described [32]. Briefly, poly-HEMA was dissolved in 95% ethanol at 12 mg/ml and applied to Petri dishes at 0.95 μl/mm^2^. Coated dishes were washed twice with PBS before use.

### 5.13. RT-qPCR analysis

The relative mRNA levels of MMP9 were assessed by RT-qPCR in HK-2 and 786 O cell lines. mRNA was extracted using RNA Trizol (Invitrogen) and reverse-transcribed into cDNA with the PrimeScrip RT reagent kit (Takara). The ChamQ Universal SYBR^®^ qPCR Master Mix (Vazyme) was employed to prepare the RT-qPCR mixture. The mixture was then detected and analyzed on a Roche LightCycler^®^ 96, with β-actin serving as an internal control. Primers used in this study are listed in Supplementary Table S2.

### 5.14. Western blot assay

Standard protocols were followed for sample preparation, with cells lysed using RIPA buffer and protein concentrations determined *via* BCA assay. Proteins were separated on SDS-PAGE gels and transferred onto PVDF membranes. The membranes were then blocked with 5% BSA for 1 h, incubated with primary antibodies overnight at 4 °C, and secondary antibodies for 1 h at room temperature. Immunoblotting bands were visualized using an SDS-PAGE chemiluminescence detector. The antibodies used in this study include: MMP9 (Abmart, #T55205, 1:1000), β-Actin (Proteintech, #66009-1-Ig, 1:5000), GAPDH (Proteintech, #60004-1-Ig, 1:5000), Cleaved Caspase 3 (CST, #9664S, 1:1000), anti-Mouse secondary antibody (Proteintech, Cat: #SA00001-1, 1:5000) and anti-Rabbit secondary antibody (Proteintech, Cat: #SA00001-2, 1:5000).

### 5.15. Preparation of plasmids

The pLKO.1 plasmid, obtained from Miaolingbio, was used as shNC. The shMMP9 plasmid was constructed based on the backbone of the pLKO.1 plasmid, with the shRNA sequence for MMP9 designed by the GPP Web Portal (portals.broadinstitute.org). The primers used in this study are listed in Supplementary Table S2.

### 5.16. Flow cytometric analysis

After treatment, cells were digested by EDTA-free trypsin and washed twice with PBS. The cells were then resuspended in binding buffer and stained with Annexin V and DAPI for 10 min to detect apoptosis. All samples were analyzed using a CyAn bench-top analyzer (Beckman Coulter).

### 5.17. Wound-healing assay

Cells were seeded in 6-well plates and multiple parallel scratches were made using a 1 ml pipette tip when cell confluence reached 90% to 100%. Serum-free medium was then added to continue culturing the cells. Images were captured using an inverted microscope, and wound closure was analyzed using Fiji software (a distribution of ImageJ2) [105].

### 5.18. Transwell invasion assay

The invasive capacity of RCC cells was assessed using Transwell chambers (8-μm pore size; Corning, USA) pre-coated with Matrigel (Corning, USA) to simulate the extracellular matrix. Cells were serum-starved for 12 h, resuspended in serum-free medium, and seeded into the upper chamber at a density of 30,000 cells per well. The lower chamber was filled with complete medium containing 10% FBS as a chemoattractant. After 48 h of incubation at 37 °C, non-invading cells on the upper membrane surface were carefully removed with a cotton swab. Invaded cells on the lower surface of the membrane were fixed with 4% paraformaldehyde, stained with 0.1% crystal violet, and imaged under an inverted microscope. The number of invaded cells was quantified using Fiji software [105].

### 5.19. Statistical analysis

Statistical analyses and data visualization were conducted using R software (version 4.1.0) and Python (version 3.9.7), while experimental data were analyzed using Prism 9 (version 9.4.1) and Fiji (version 2.9.0). Measurement data are presented as mean ± SD, and Student’s t-test or one-way ANOVA was employed for appropriate data comparisons. A *p*-value < 0.05 was considered statistically significant. *, **, *** and **** denote *p* < 0.05, *p* < 0.01, *p* < 0.001 and *p* < 0.0001, respectively.

## Supplementary Material

Supplemental Material

Supplementary Table S2.docx

Supplementary Figure 2.tif

Supplementary Table S1.xlsx

Supplementary Figure 4.tif

Supplementary Figure 6.tif

Supplementary Figure 1.tif

Supplementary Figure 5.tif

Supplementary Figure 7.tif

Supplementary Figure 3.tif

## Data Availability

All RNA-seq data analyzed in our study are available in UCSC Xena (http://xena.ucsc.edu/), the Gene Expression Omnibus (https://www.ncbi.nlm.nih.gov/geo/) and the ArrayExpress database (https://www.ebi.ac.uk/biostudies/arrayexpress). Other data and code are available upon reasonable request from the corresponding author, Bin Fu or Jianhan Fu.
