# Supplementary material for: Anoikis-related genes predicts prognosis and therapeutic response in renal cell carcinoma
Source: Ann Med. 2025 Aug 19;57(1):2548042. doi: 10.1080/07853890.2025.2548042 (PMC12366518; doi:10.1080/07853890.2025.2548042)
Supplement: Supplemental Material [file IANN_A_2548042_SM0550.docx]

**Supplementary Table S1. Anoikis-related genes**

(No additional legend required; the title fully describes the content.)

**Supplementary Table S2. The incorporated primers.**

qPCR^a^: Quantitative polymerase chain reaction

MMP9^b^: Matrix metallopeptidase 9

KD^c^: Knockdown

**Supplementary Figure 1.** ABCD using machine learning methods to screen robust signature genes.

(No additional legend required; the title fully describes the content.)

**Supplementary Figure 2.** The distribution of clinical features between two groups.

(No additional legend required; the title fully describes the content.)

**Supplementary Figure 3.** Volcano plot of the DEGs between two groups.

(No additional legend required; the title fully describes the content.)

**Supplementary Figure 4.** Summarized the mutation profiles of two groups.

(No additional legend required; the title fully describes the content.)

**Supplementary Figure 5.** A & B Quality control process; C. Elbowplot indicated the finest component numbers; D. UAMP plot shows the batch effect had been removed; E – G. Annotation of celltype.

**Supplementary Figure 6.** A. Top target genes in ARGs_high tumor cells and top ligands in T cells; B. Top target genes in ARGs_low tumor cells and top ligands in T cells.

**Supplementary Figure 7.** A. Venn plot shows the MMP9 as the candidate genes; B. K-M plot of O; C. K-M plot of DFS; D. MMP9 mRNA expression levels between tumor and normal samples; E. MMP9 mRNA expression levels between primary and metastasis tumors; F. MMP9 mRNA expression levels between ARGs-high and -low groups.
