# Supplementary material for: Anoikis-related genes predicts prognosis and therapeutic response in renal cell carcinoma
Source: Ann Med. 2025 Aug 19;57(1):2548042. doi: 10.1080/07853890.2025.2548042 (PMC12366518; doi:10.1080/07853890.2025.2548042)
Supplement: Supplementary Table S2.docx [file IANN_A_2548042_SM4305.docx]

**Supplemental Table S2. The incorporated primers.**

| **Primer name** | **Sequence 5'—3'** |
| --- | --- |
| **β-actin-qPCR^a^-F** | CATGTACGTTGCTATCCAGGC |
| **β-actin-qPCR-R** | CTCCTTAATGTCACGCACGAT |
| **MMP9^b^-qPCR-F** | TCGTGGTTCCAACTCGGTTT |
| **MMP9-qPCR-R** | TCCTGGGTGTAGAGTCTCTCG |
| **MMP9-KD^c^-F** | CCGGCATTCAGGGAGACGCCCATTTCTCGAGAAATGGGCGTCTCCCTGAATGTTTTTG |
| **MMP9-KD-R** | AATTCAAAAACATTCAGGGAGACGCCCATTTCTCGAGAAATGGGCGTCTCCCTGAATG |

qPCR^a^: Quantitative polymerase chain reaction

MMP9^b^: Matrix metallopeptidase 9

KD^c^: Knockdown
