# Supplementary figures and images for: Anoikis-related genes predicts prognosis and therapeutic response in renal cell carcinoma
Source: Ann Med. 2025 Aug 19;57(1):2548042. doi: 10.1080/07853890.2025.2548042 (PMC12366518; doi:10.1080/07853890.2025.2548042)

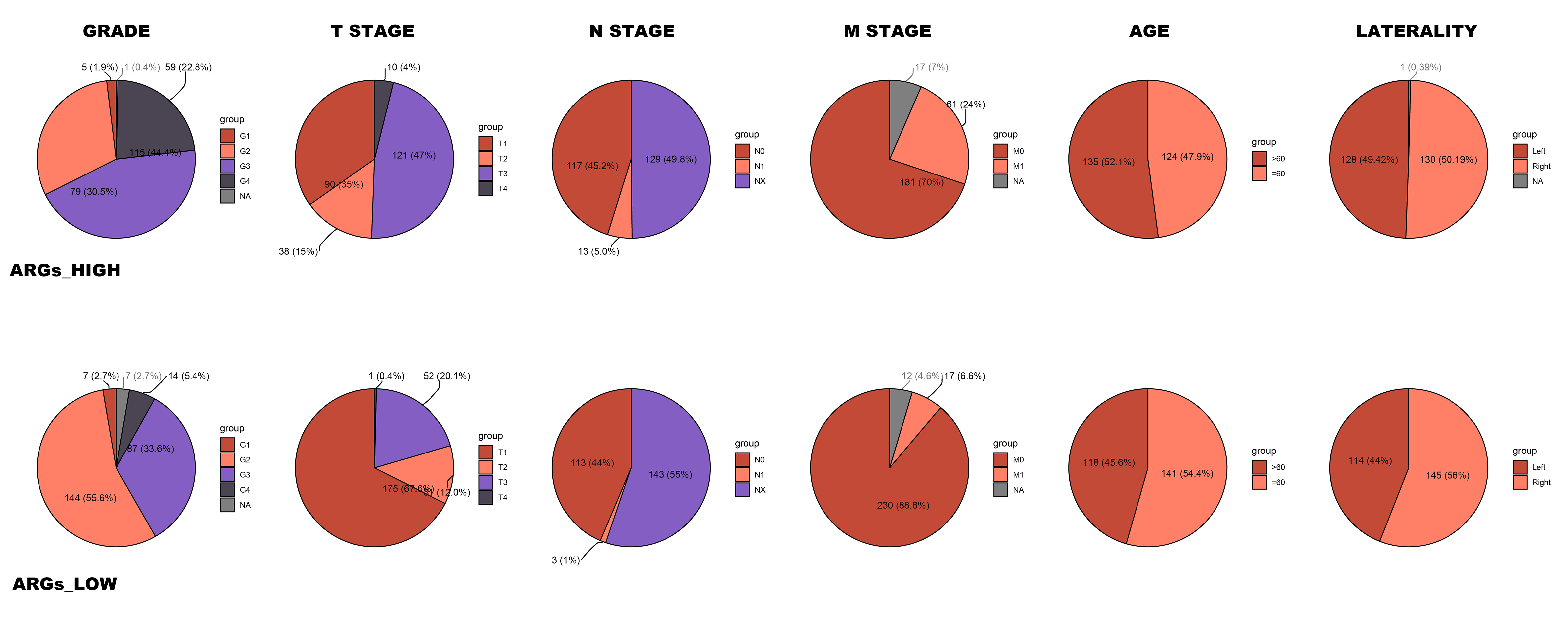

Supplement: Supplementary Figure 2.tif [file IANN_A_2548042_SM4304.tif]

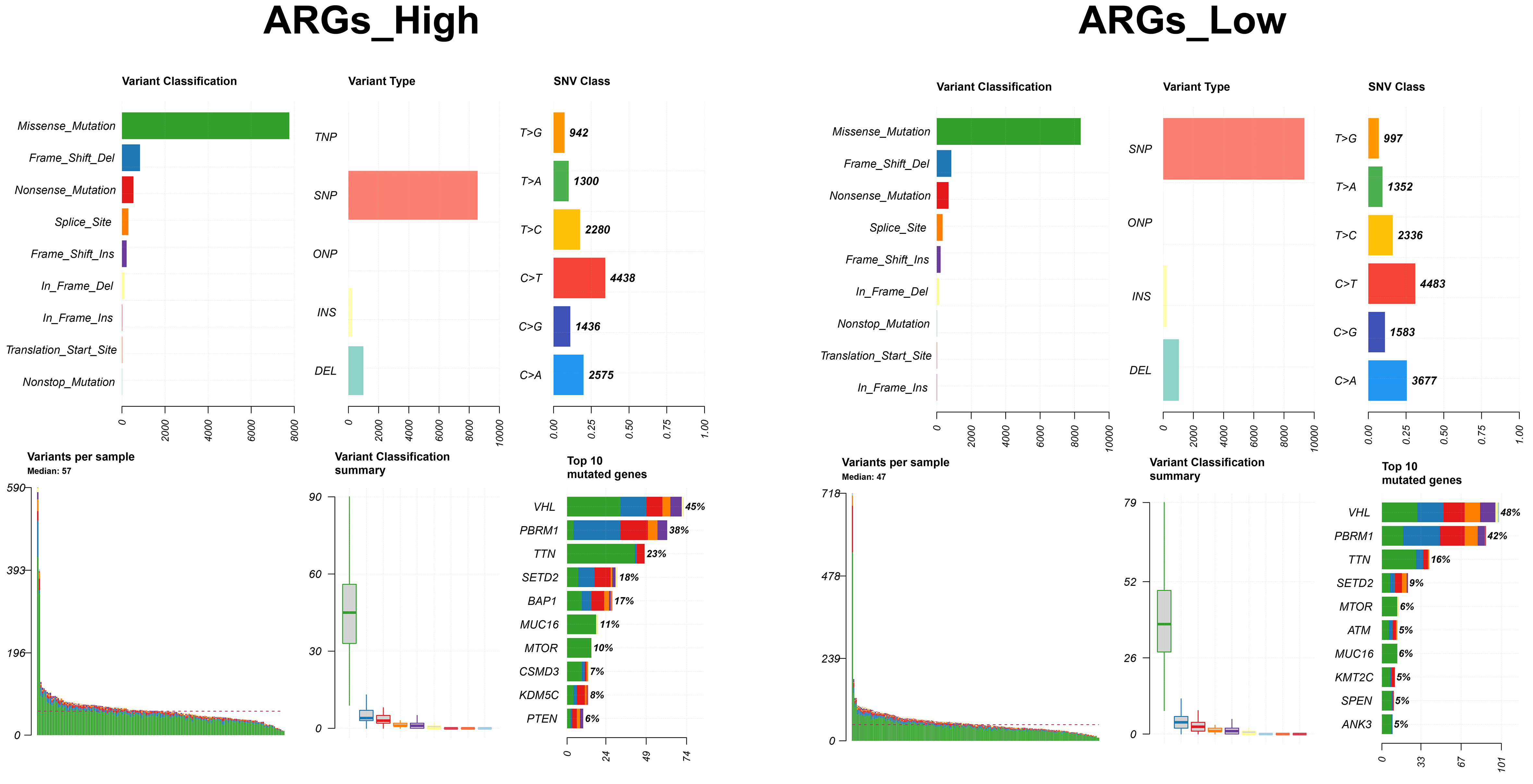

Supplement: Supplementary Figure 4.tif [file IANN_A_2548042_SM4302.tif]

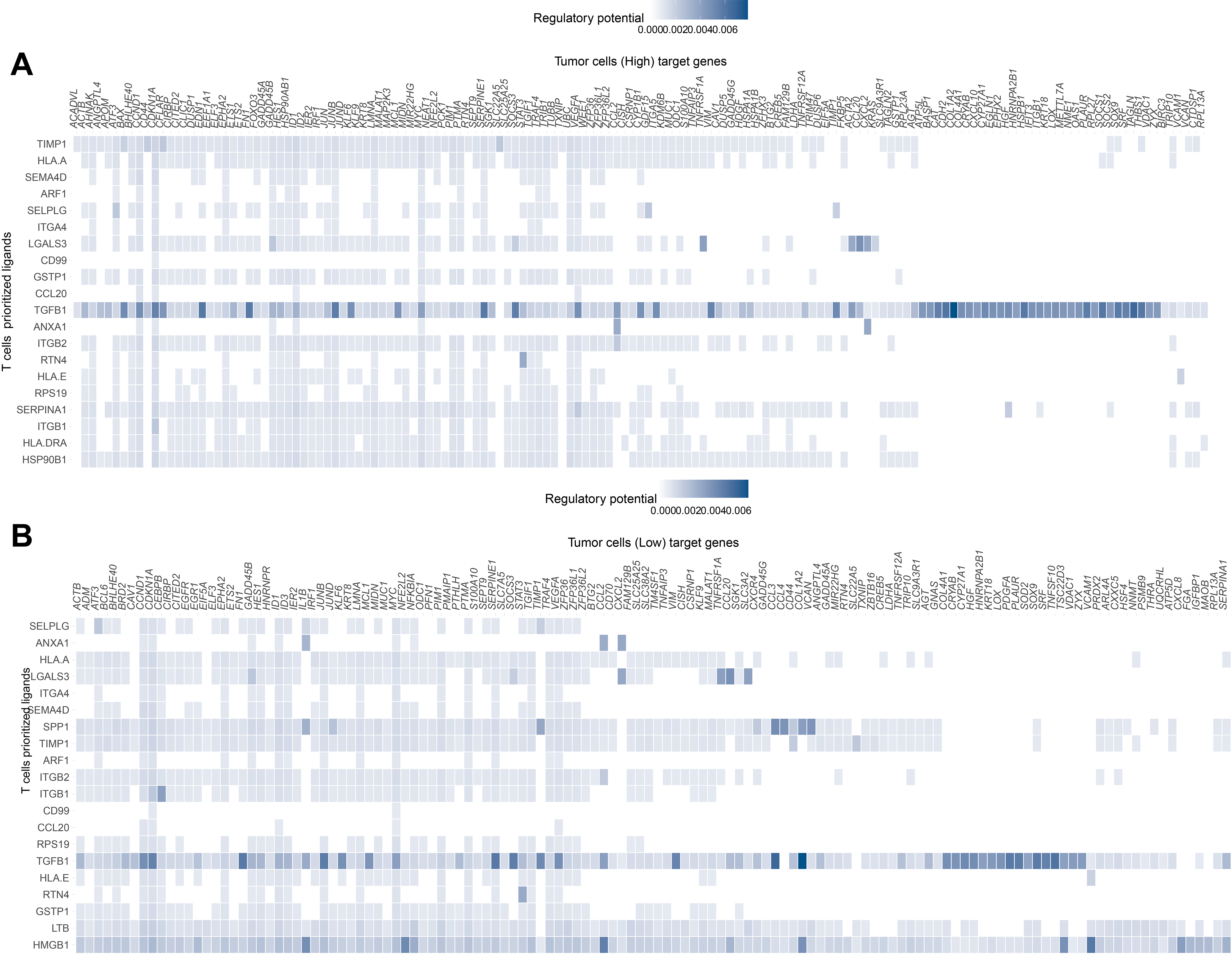

Supplement: Supplementary Figure 6.tif [file IANN_A_2548042_SM4301.tif]

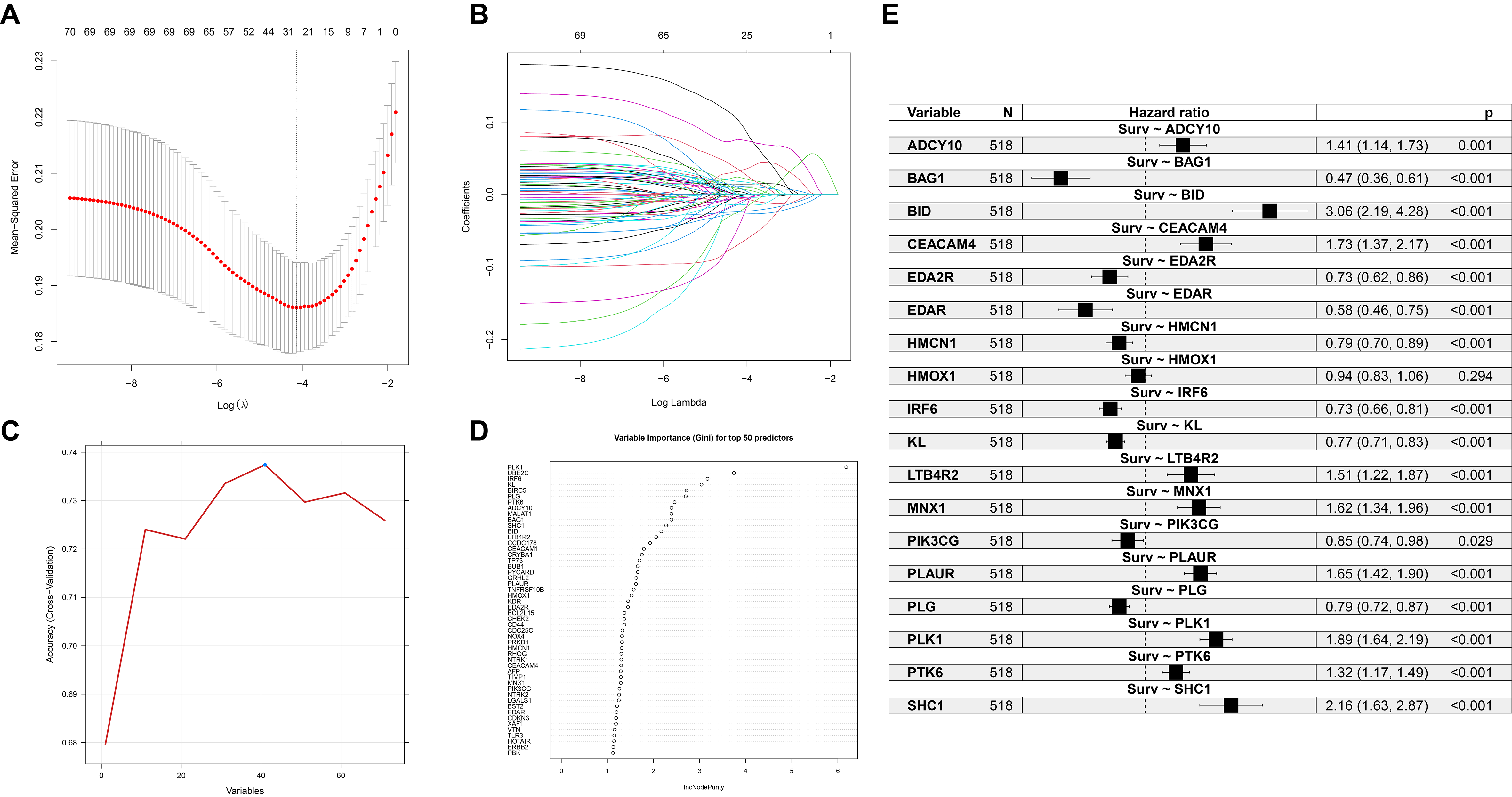

Supplement: Supplementary Figure 1.tif [file IANN_A_2548042_SM4300.tif]

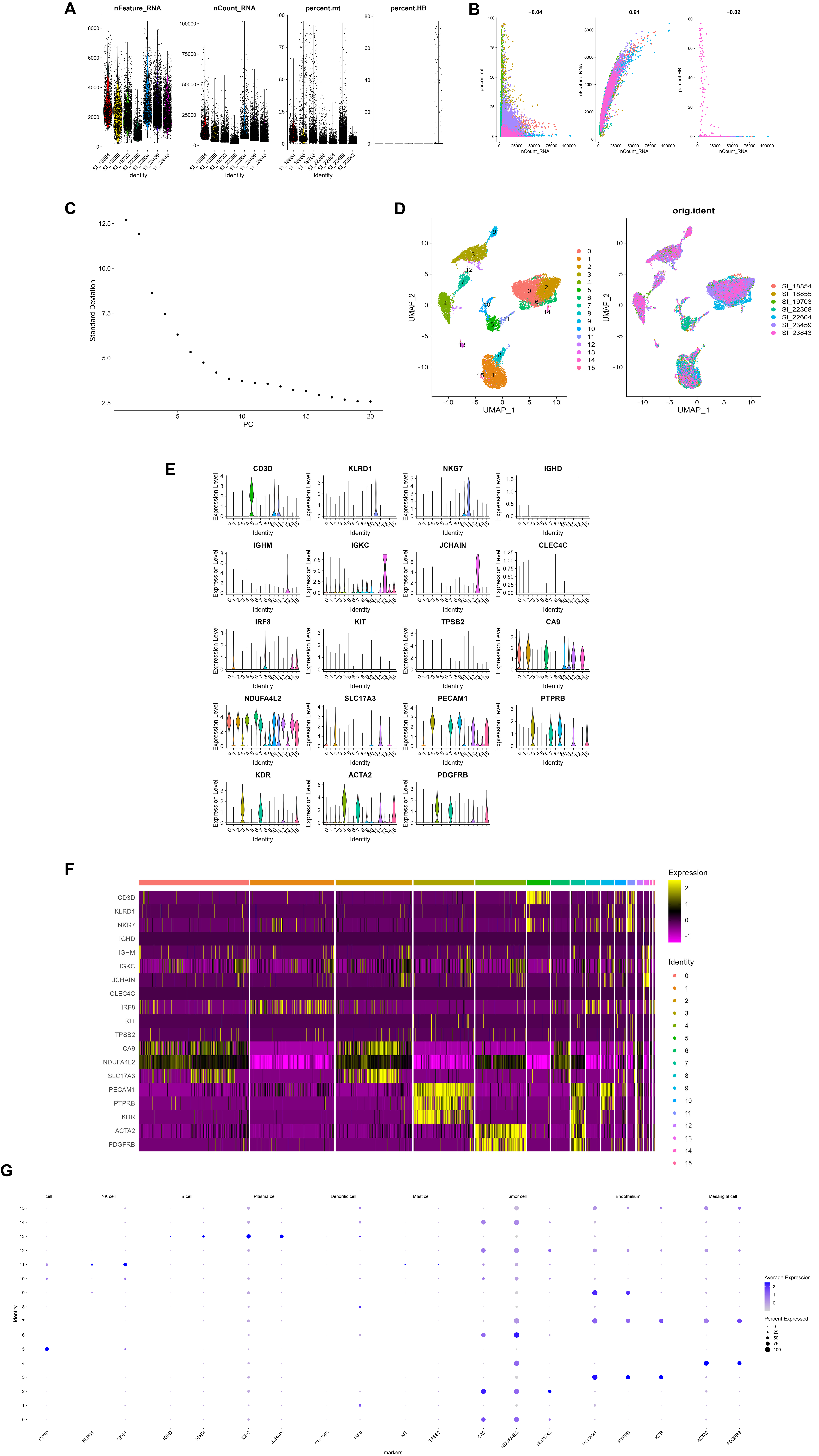

Supplement: Supplementary Figure 5.tif [file IANN_A_2548042_SM4299.tif]

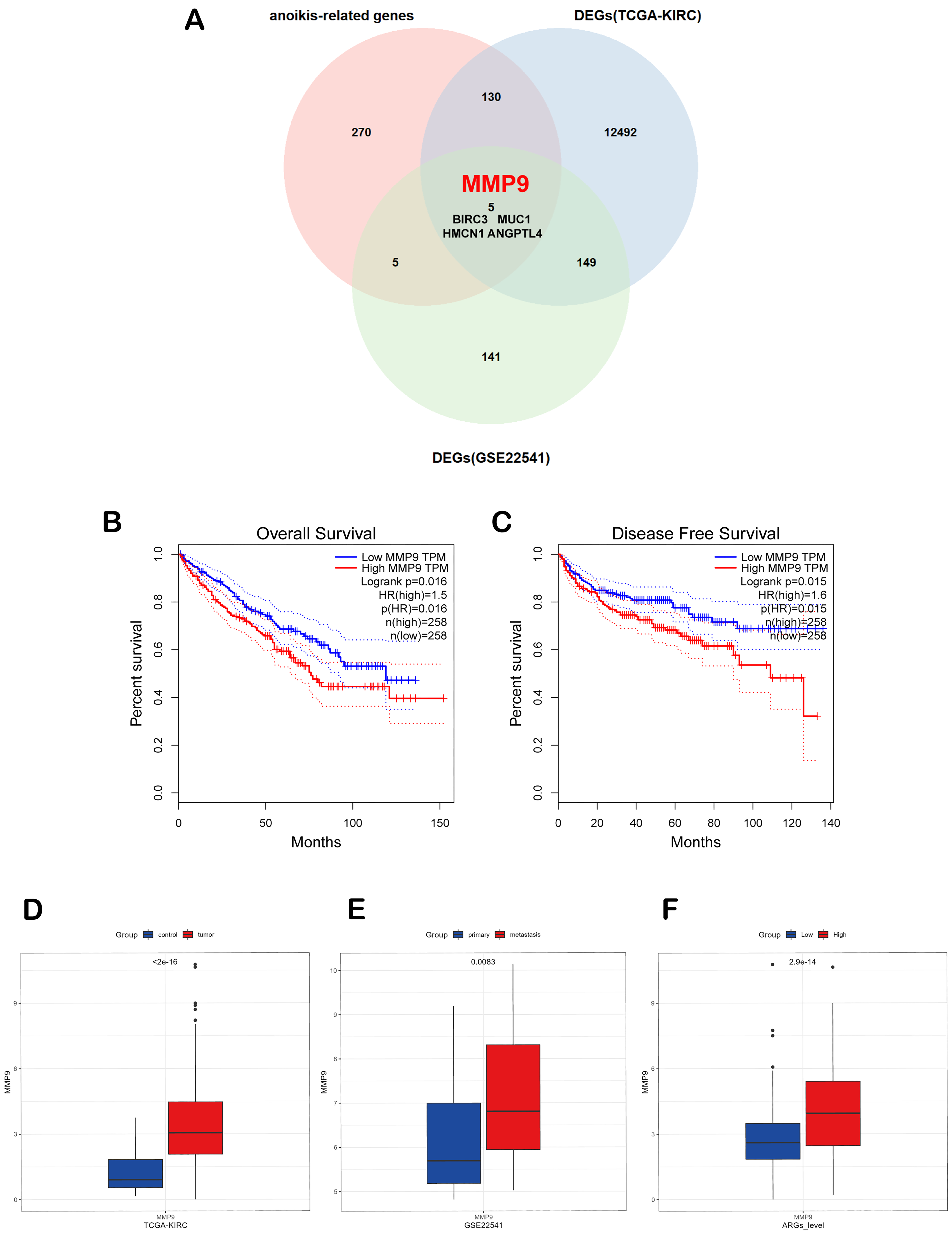

Supplement: Supplementary Figure 7.tif [file IANN_A_2548042_SM4298.tif]

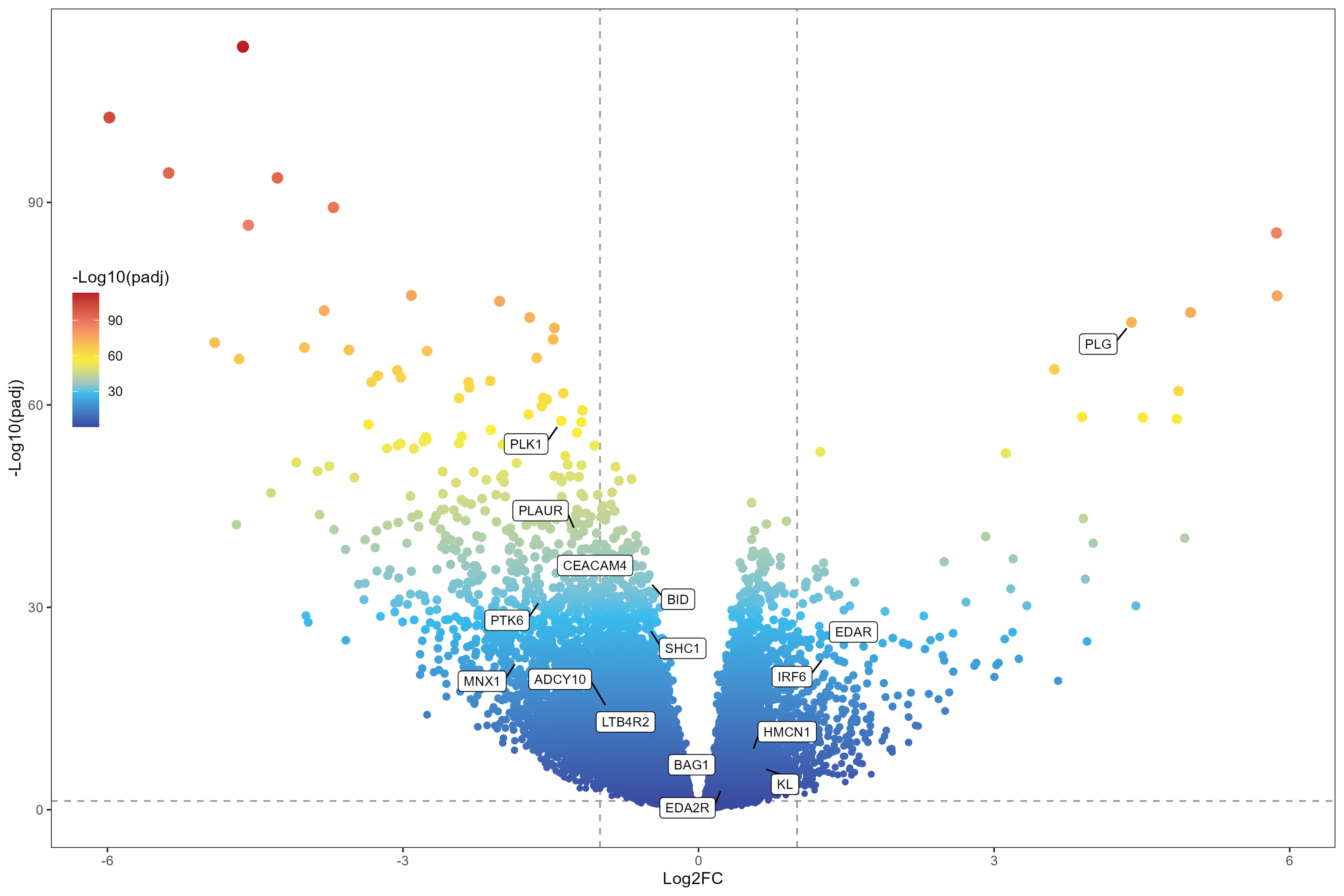

Supplement: Supplementary Figure 3.tif [file IANN_A_2548042_SM4296.tif]
